# Early Recurrence of an Adult Desmoplastic Medulloblastoma With Intramedullary Spinal and Suprasellar Metastases

**DOI:** 10.7759/cureus.106018

**Published:** 2026-03-28

**Authors:** Elizabeth Blanco Espinosa, Idania Cruzata Matos, Ricardo Marlon Saro Del Valle, Francisco J Santana Mercedes, Siurys D Mata Rieumont, Yulio L Aguila

**Affiliations:** 1 General Practice, CEDA Orthopedic Group, Miami, USA; 2 General Surgery, Hospital Arnaldo Milian, Santa Clara, CUB; 3 General Medicine, HCA Healthcare, Las Vegas, USA; 4 Neurology, Hospital General Dr. Juan Bruno Zayas Alfonso, Santiago de Cuba, CUB; 5 Geriatrics, Universidad Central del Este, Egg Harbor City, USA; 6 Medicine, Universidad de Ciencias Médicas, Ciego de Ávila, CUB; 7 Primary Care - Research Initiative (PCRI), Adtremed Research Clinic, Land O' Lakes, USA

**Keywords:** adult medulloblastoma, craniospinal irradiation, intramedullary metastasis, recurrence, suprasellar metastasis

## Abstract

Medulloblastoma is a highly aggressive embryonal tumor of the central nervous system with a strong tendency for dissemination through cerebrospinal fluid. While it occurs predominantly in pediatric populations, it is uncommon in adults. Intramedullary spinal metastases and suprasellar involvement are exceptionally rare patterns of dissemination in adults. We report the case of a 21-year-old male diagnosed with desmoplastic medulloblastoma of the cerebellar vermis who underwent ventriculoperitoneal shunting, gross total resection, and craniospinal radiotherapy. Despite receiving standard multimodal therapy, the patient developed recurrence within 12 months, presenting with rapidly progressive paraplegia and bilateral blindness. Magnetic resonance imaging (MRI) revealed tumor recurrence in the posterior fossa, suprasellar metastases involving the optic chiasm, and extensive spinal dissemination, including intramedullary and intradural metastatic lesions. This case highlights the potential for early recurrence and extensive neuroaxis dissemination in adult medulloblastoma despite initially favorable histopathology and appropriate multimodal therapy. The coexistence of intramedullary spinal metastases and suprasellar involvement represents an exceptionally rare dissemination pattern in adults, underscoring the importance of maintaining a high index of suspicion during follow-up. The development of new neurological deficits during follow-up should prompt urgent imaging of the entire neuroaxis to evaluate for metastatic dissemination. Comprehensive neuraxis surveillance and molecular characterization remain critical components in the evaluation and management of adult medulloblastoma, as they provide essential information for prognostic assessment and therapeutic decision-making.

## Introduction

Medulloblastoma is a highly malignant embryonal tumor of the cerebellum classified as WHO grade 4 and characterized by aggressive biological behavior and a marked propensity for cerebrospinal fluid dissemination [1]. Although it represents the most common primary malignant brain tumor in children, its occurrence in adults is distinctly uncommon, accounting for less than 1% of primary intracranial neoplasms [2]. Population-based data estimate an annual incidence of approximately 0.5-0.6 cases per million adults, markedly lower than that observed in pediatric populations [2].

Adult medulloblastoma most frequently presents in the third to fourth decades of life and demonstrates a slight male predominance. Importantly, adult tumors exhibit distinct molecular and biological characteristics compared with pediatric disease, with Sonic Hedgehog (SHH)-activated tumors representing the predominant molecular subgroup in adults [3]. These molecular subtypes are associated with distinct clinical behaviors, recurrence patterns, and prognostic outcomes, which are essential for risk stratification in adult patients.

The peak incidence of medulloblastoma occurs in early childhood, most commonly between two and six years of age, with a consistent male predominance [3]. In pediatric patients, tumors typically arise in the midline cerebellar vermis, whereas adult medulloblastomas more frequently originate in the cerebellar hemispheres [3]. Clinically, patients often present with symptoms of increased intracranial pressure secondary to obstruction of the fourth ventricle and hydrocephalus, including headache, nausea, vomiting, and papilledema, frequently accompanied by gait ataxia and other cerebellar deficits [3]. Recognition of these age-related anatomical distinctions is essential when evaluating posterior fossa tumors in adults.

A defining feature of medulloblastoma is its tendency to disseminate through the cerebrospinal fluid, a characteristic that significantly influences staging, prognosis, and therapeutic strategy [3]. Tumor cells commonly spread along leptomeningeal surfaces, resulting in so-called “drop metastases” that predominantly involve the spinal subarachnoid space, particularly the lumbosacral region [3].

Metastatic disease is present at diagnosis in approximately 20-30% of pediatric cases and in a smaller but clinically meaningful proportion of adults [2,3]. However, intramedullary spinal metastases are rare and have been described only in limited case reports and small case series, while suprasellar dissemination is also exceptionally uncommon in adults, with only sporadic cases reported in the literature. These atypical dissemination patterns are often associated with advanced disease and may indicate a more unfavorable clinical course.

Management of adult medulloblastoma requires a multidisciplinary approach centered on maximal safe surgical resection followed by risk-adapted craniospinal irradiation [4]. The role of adjuvant chemotherapy is individualized according to molecular subgroup, performance status, and metastatic staging [4]. International clinical practice guidelines emphasize that therapeutic decisions in adults must account for differences in tumor biology, treatment tolerance, and long-term neurological sequelae compared with pediatric populations [4].

Despite multimodal therapy, recurrence remains a significant clinical challenge, particularly when metastatic spread occurs in uncommon anatomical locations. To date, reports describing the simultaneous occurrence of intramedullary spinal metastases and suprasellar involvement in adult medulloblastoma are exceedingly limited, and their presentation in the setting of early recurrence has not been well characterized in the literature.

To our knowledge, the simultaneous occurrence of intramedullary spinal metastases and suprasellar involvement in early recurrence of adult medulloblastoma has been described only rarely, further emphasizing the clinical relevance of this presentation. Here, we report a case of early recurrence in adult desmoplastic medulloblastoma with simultaneous posterior fossa, suprasellar, and intramedullary spinal metastases. This case highlights a rare and complex dissemination pattern and underscores the importance of comprehensive neuroaxis surveillance during follow-up.

## Case presentation

History and clinical findings

A 21-year-old previously healthy male presented with a one-year history of progressively worsening occipital headache, morning-predominant vomiting, blurred vision, and gait instability. Over the preceding three months, he reported worsening imbalance and intermittent diplopia. There was no history of seizures, prior malignancy, or familial cancer syndromes. On admission, neurological examination revealed bilateral papilledema on fundoscopic examination, horizontal nystagmus, truncal ataxia with a wide-based gait, mild dysmetria on finger-to-nose testing, intact motor strength (5/5) in all extremities, normal muscle tone, preserved deep tendon reflexes, and no sensory deficits. The cranial nerve examination demonstrated intact extraocular movements with intermittent diplopia, no facial asymmetry, preserved hearing, and normal gag reflex. Visual acuity was reduced bilaterally (20/60), with preserved pupillary light reflexes at presentation. The Glasgow Coma Scale was 15/15. Vital signs were stable. The clinical presentation was consistent with increased intracranial pressure secondary to obstructive hydrocephalus.

Imaging findings

**Figure 1:**
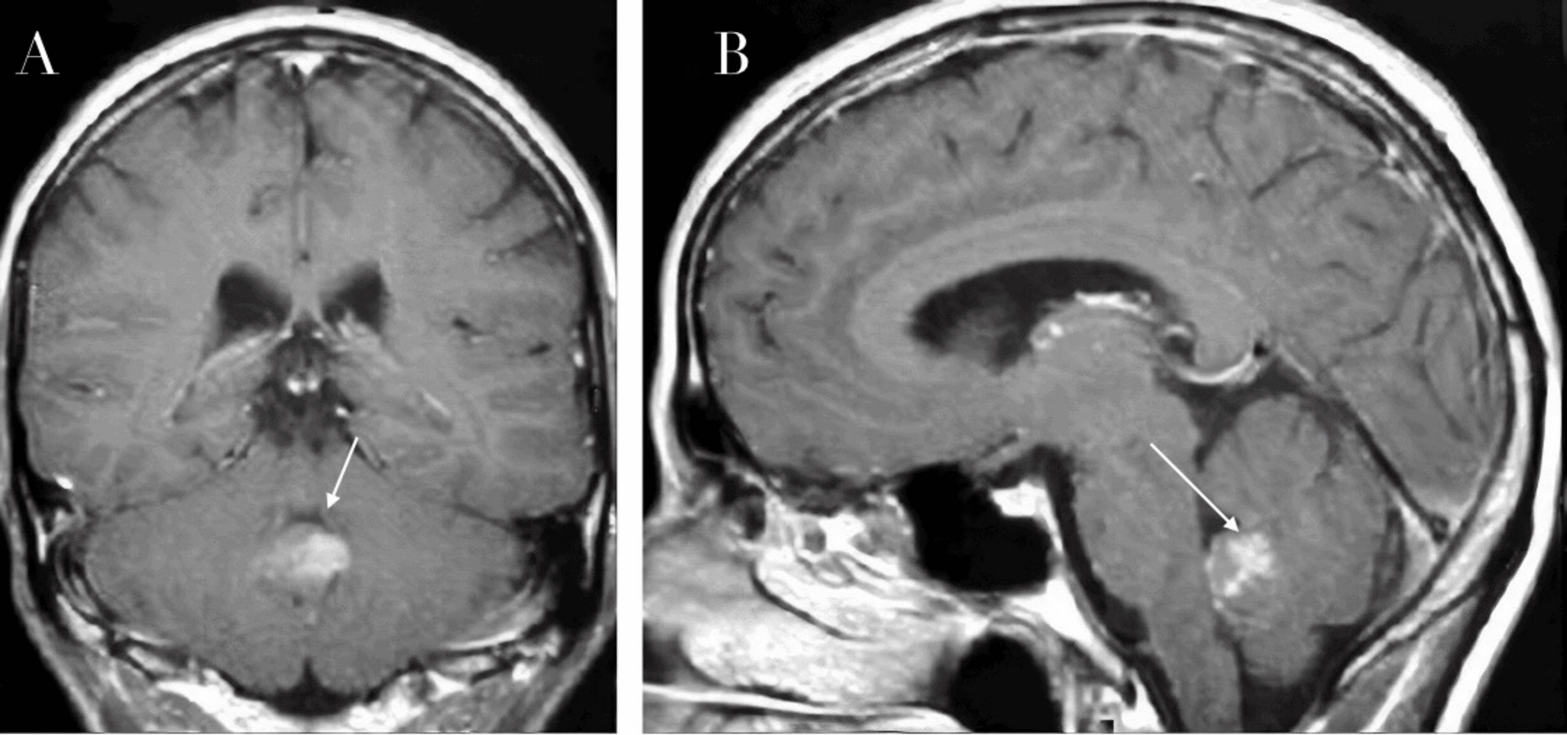
Initial contrast-enhanced brain MRI obtained at hospital admission. A: Coronal T1-weighted post-contrast image demonstrating a heterogeneously enhancing midline posterior fossa mass (white arrow) centered in the cerebellar vermis, measuring approximately 4.2 × 3.8 × 3.5 cm. The lesion shows avid, irregular contrast enhancement. There is marked compression and anterior displacement of the fourth ventricle, resulting in obstructive triventricular hydrocephalus. B: Sagittal T1-weighted post-contrast image confirming a heterogeneously enhancing mass centered in the cerebellar vermis (white arrow). The lesion causes a significant mass effect on the fourth ventricle, with anterior displacement and obstructive hydrocephalus. No evidence of spinal leptomeningeal dissemination was identified on initial staging MRI of the neuroaxis.

Brain MRI demonstrated a midline posterior fossa mass centered in the cerebellar vermis, measuring approximately 4.2 × 3.8 × 3.5 cm, with a hypointense signal on T1-weighted imaging and heterogeneous hyperintensity on T2-weighted imaging. Diffusion-weighted imaging (DWI) demonstrated marked restricted diffusion with corresponding low apparent diffusion coefficient (ADC) values, consistent with a hypercellular tumor such as medulloblastoma. Intense heterogeneous contrast enhancement and compression and anterior displacement of the fourth ventricle. Obstructive triventricular hydrocephalus. No spinal lesions were identified on the initial neuroaxis staging MRI (Figure 1).

Surgical procedure

Due to symptomatic hydrocephalus, the patient initially underwent ventriculoperitoneal shunt placement, resulting in symptomatic improvement. One week later, a midline suboccipital craniectomy via a transvermian approach was performed under general anesthesia. Intraoperatively, the tumor appeared grayish and moderately vascular, firm with partially infiltrative margins, and adherent to the inferior vermis. Microsurgical dissection allowed gross total resection, confirmed by postoperative MRI within 48 hours. No intraoperative complications occurred. Estimated blood loss was approximately 350 mL. Postoperatively, the patient was monitored in the neurosurgical intensive care unit for 48 hours. He remained neurologically intact except for mild residual gait ataxia.

Histopathological findings

Histological examination revealed a highly cellular tumor composed of small round blue cells, hyperchromatic nuclei with scant cytoplasm, frequent mitotic figures, nodular architecture with reticulin-rich desmoplastic stroma, and Homer-Wright pseudorosettes. Immunohistochemistry showed synaptophysin positivity and a Ki-67 proliferative index of approximately 35%. The findings confirmed desmoplastic/nodular medulloblastoma (WHO grade 4). Molecular subgroup analysis was not available due to resource limitations.

Adjuvant therapy

The patient received craniospinal irradiation (36 Gy) with a posterior fossa boost to 54 Gy, delivered over six weeks. Adjuvant chemotherapy was not administered due to institutional protocol limitations and patient preferences after multidisciplinary discussion. It is important to note that combined chemotherapy is commonly recommended in adult medulloblastoma, and omission of chemotherapy may influence disease control.

**Figure 2:**
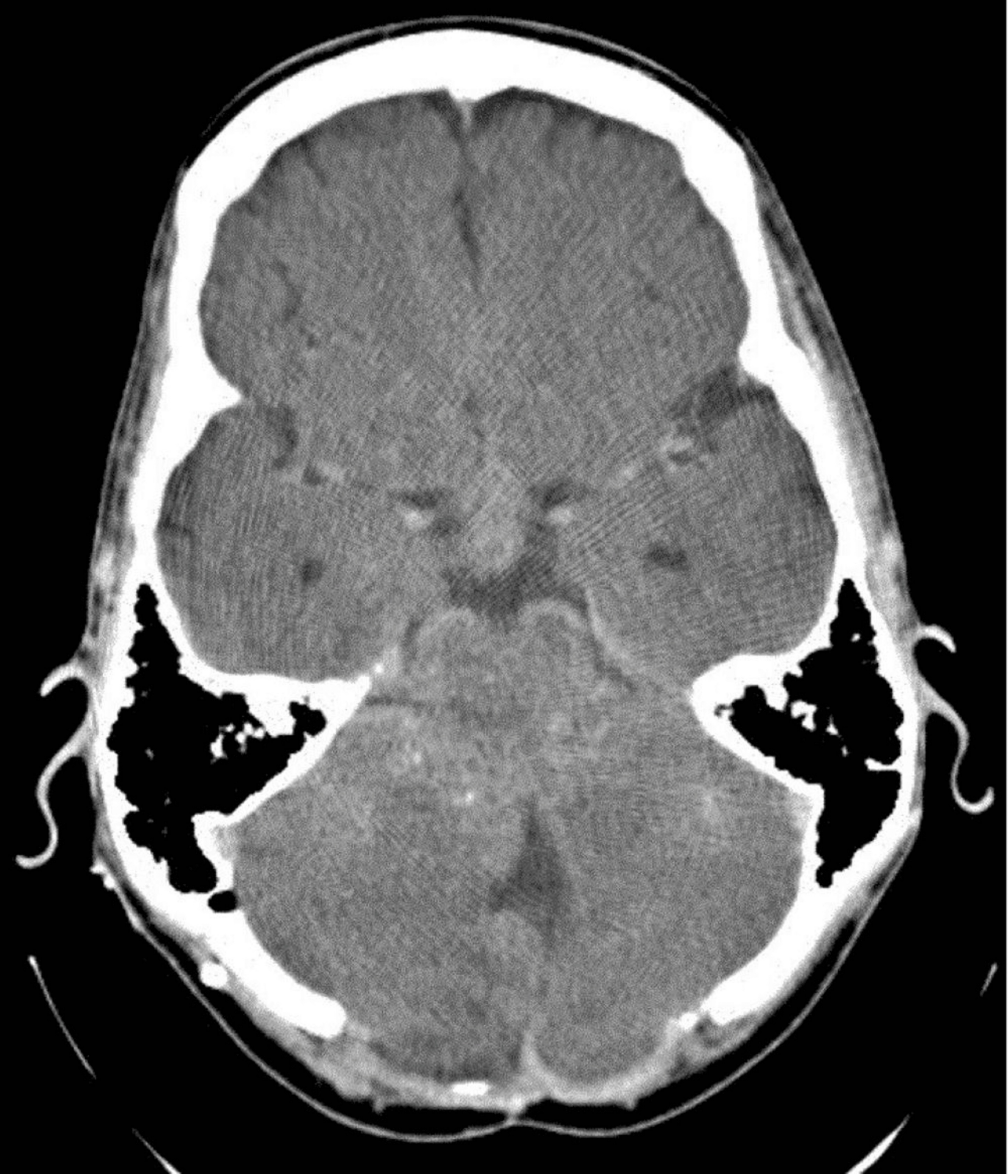
Follow-up brain CT performed six months after surgery.

At the six-month follow-up, contrast-enhanced CT demonstrated postoperative changes without evidence of recurrence (Figure 2).

Recurrence and follow-up

Twelve months after surgery, the patient presented with progressive bilateral lower extremity weakness, numbness below the T6 dermatome, and declining visual acuity. Within one week, neurological deterioration progressed to complete paraplegia (Medical Research Council grade 0/5), sensory level at T6, hyperreflexia and bilateral Babinski signs, and severe bilateral visual loss progressing to near-complete blindness, with non-reactive pupils, suggesting advanced optic pathway involvement beyond isolated chiasmal compression.

Brain MRI performed one year after surgery demonstrated postoperative changes consistent with prior suboccipital craniectomy. Imaging sequences included axial proton density, FLAIR, and T2-weighted images, as well as sagittal T1- and T2-weighted sequences with gadolinium enhancement. A heterogeneously enhancing soft tissue mass was identified in the posterior fossa, located between the posterior surface of the brainstem and the anterior aspect of the cerebellar hemispheres, occupying the surgical bed. The lesion measured approximately 3.5 cm in maximal diameter and demonstrated compression and anterior displacement of the fourth ventricle, effacement of the perimesencephalic cisterns, direct infiltration and compression of the dorsal brainstem, and angulation and distortion of the distal medulla oblongata. The mass showed heterogeneous hyperintensity on T2-weighted imaging and avid contrast enhancement, consistent with tumor recurrence.

**Figure 3:**
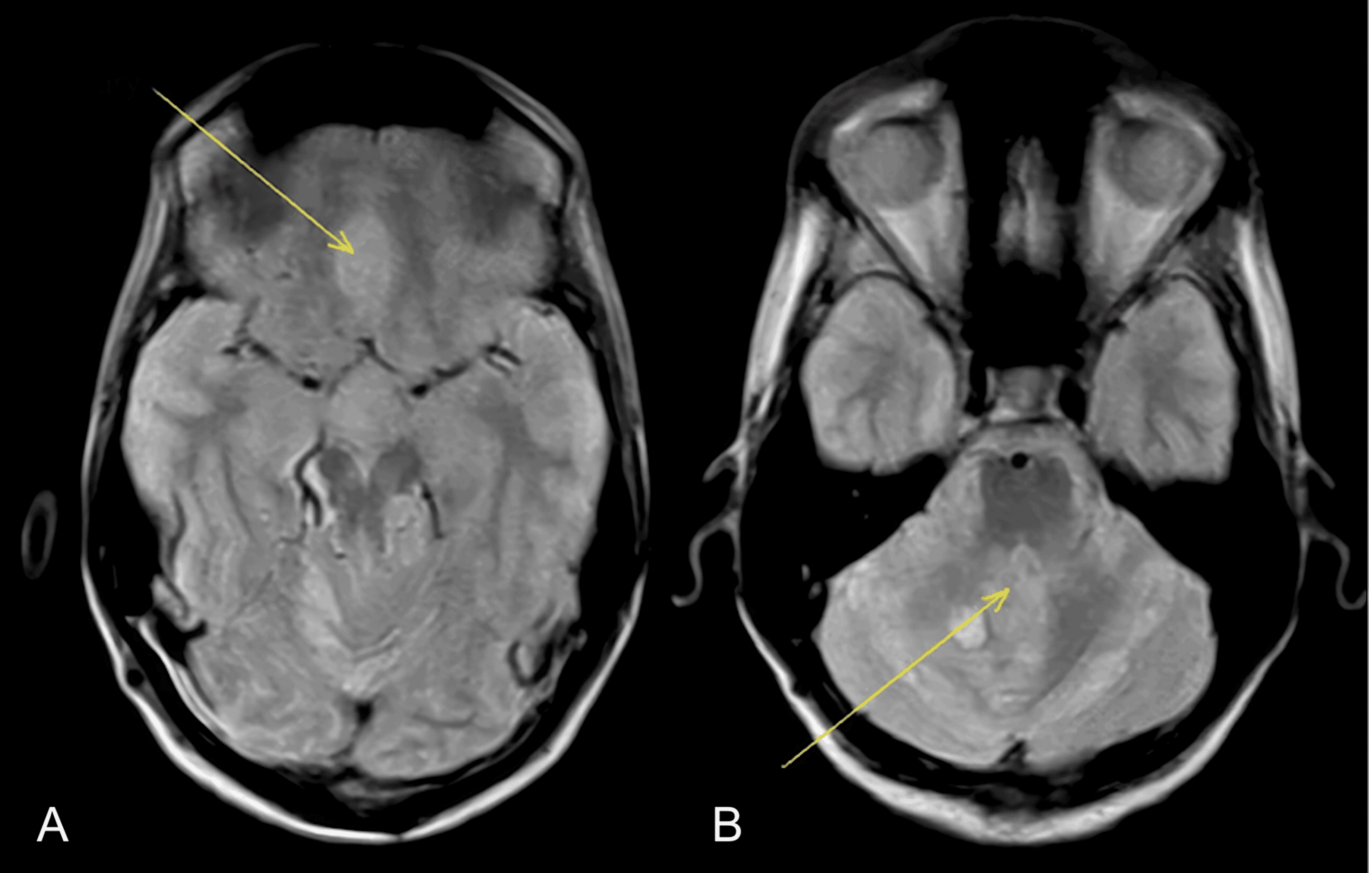
Brain MRI performed one year after surgery. Axial brain MRI demonstrating recurrent posterior fossa tumor and suprasellar metastatic involvement. A: Heterogeneously enhancing mass centered in the posterior fossa with mass effect on the cerebellum and brainstem (yellow arrow). B: Suprasellar enhancing lesion with involvement of the optic chiasm (yellow arrow), consistent with intracranial metastatic dissemination.

Additionally, multiple nodular enhancing lesions were observed along the basal surfaces of the frontal lobes and within the suprasellar cistern. The suprasellar lesion demonstrated encasement of the optic chiasm, correlating clinically with the patient’s bilateral visual loss. Mild asymmetric dilation of the lateral ventricles was noted, without evidence of acute obstructive hydrocephalus. These findings were consistent with extensive intracranial metastatic dissemination (Figure 3).

Cervical and thoracic spine MRI findings

MRI was performed using sagittal T1- and T2-weighted sequences, axial and coronal T2-weighted images, and post-contrast T1-weighted sequences. Vertebral body height, alignment, and marrow signal intensity were preserved. Intervertebral discs appeared normal, with no evidence of degenerative or compressive pathology. However, abnormal findings were identified within the spinal cord: an expansile intramedullary lesion extending from C6 to approximately T4, diffuse cord enlargement over this segment, heterogeneous hyperintensity on T2-weighted sequences, and patchy and nodular contrast enhancement on post-gadolinium T1-weighted images. The spinal cord contour appeared irregular and lobulated in the affected region, with a loss of normal gray-white matter differentiation. Surrounding T2 hyperintensity suggested associated vasogenic edema. In addition, multiple small enhancing nodular lesions were observed along the dorsal and ventral leptomeningeal surfaces, consistent with intradural extramedullary metastatic deposits. No evidence of epidural disease or vertebral metastasis was identified.

**Figure 4:**
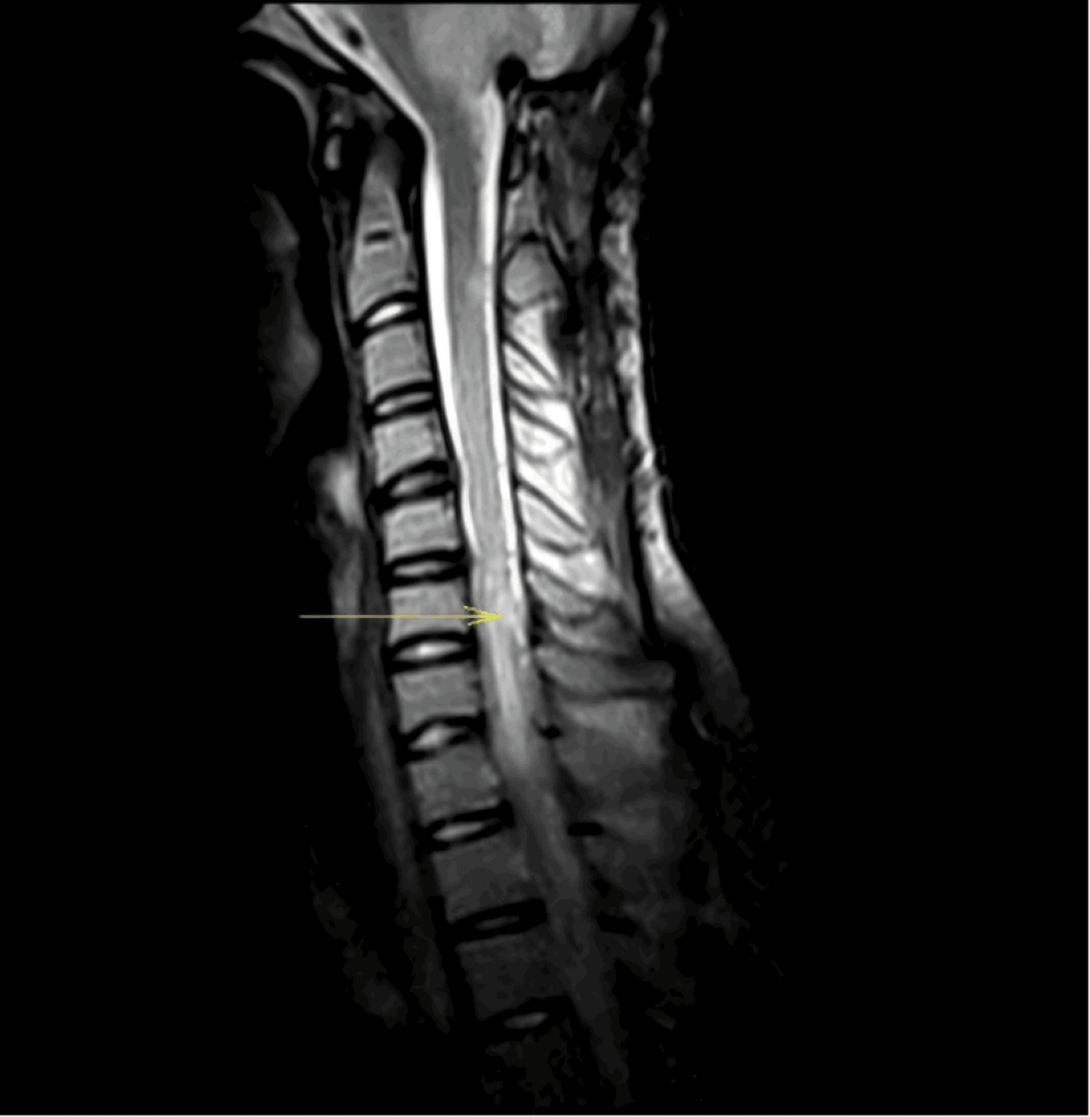
Sagittal MRI of the cervicothoracic spine demonstrating intramedullary metastatic infiltration (yellow arrow) with leptomeningeal dissemination one year after surgery.

These imaging findings were consistent with intramedullary metastatic infiltration of the cervical and upper thoracic spinal cord. Concomitant leptomeningeal dissemination (“drop metastases”) (Chang stage M3). The longitudinal extent of involvement and associated cord edema correlated clinically with the patient’s rapid progression to complete paraplegia and a T6 sensory level (Figure 4).

This recurrence pattern differs from typical adult medulloblastoma relapse, which most commonly involves leptomeningeal or spinal "drop metastases." The presence of simultaneous posterior fossa recurrence, suprasellar involvement, and intramedullary dissemination represents an atypical and extensive pattern of neuraxis spread. Key clinical warning signs in this context include rapidly progressive motor deficits, the development of a defined sensory level, and acute or subacute visual deterioration, all of which should prompt urgent MRI evaluation of the entire neuroaxis.

Ethical approval

Written informed consent was obtained from the patient and his legal representatives for publication of this case report and accompanying images. This report complies with institutional and international ethical standards. Formal institutional review board approval was not required for a single anonymized case report according to local regulations. All procedures conformed to the principles outlined in the Declaration of Helsinki.

## Discussion

Adult medulloblastoma is a rare and biologically heterogeneous malignancy that differs significantly from its pediatric counterpart in epidemiology, molecular distribution, clinical behavior, and therapeutic tolerance. Although it is the most common malignant brain tumor in children, it accounts for approximately 0.4-1% of all primary adult central nervous system tumors and less than 1% of adult intracranial neoplasms overall, with an estimated annual incidence of 0.5 cases per million and a slight male predominance [3,5]. In contrast to the early childhood peak observed in pediatric populations, adult cases most commonly present in the third and fourth decades of life.

Maximal safe resection followed by craniospinal irradiation (CSI) remains the cornerstone of treatment in adults. However, contemporary adult-focused analyses emphasize that management strategies cannot simply replicate pediatric protocols due to differences in molecular subgroup prevalence, relapse patterns, toxicity profiles, and long-term outcomes [6]. Therefore, individualized and risk-adapted approaches are recommended in adult patients.

Molecular subgrouping has substantially refined the understanding of adult medulloblastoma. Large adult cohorts confirm that SHH-activated tumors represent the predominant molecular subgroup in adulthood, accounting for approximately 60% of cases, whereas WNT and non-WNT/non-SHH (Groups 3 and 4) tumors are less frequent than in pediatric populations [1,2,6,7]. Although histologically similar across age groups, histopathological subtype alone is insufficient for prognostic stratification. Even desmoplastic/nodular tumors, commonly associated with SHH activation and traditionally considered intermediate risk, may demonstrate aggressive behavior depending on underlying molecular characteristics. Genomic analyses further demonstrate that adult medulloblastoma harbors distinct cytogenetic and mutational profiles, including frequent TERT promoter mutations and subgroup-specific chromosomal alterations [7]. Within the SHH subgroup, TP53 mutation status has emerged as a critical prognostic modifier. Within the SHH tumors, TP53 mutation status has emerged as a critical prognostic modifier, with TP53-mutated tumors associated with significantly worse outcomes [8]. A major limitation of this case is the absence of molecular profiling, which precludes precise subgroup classification and risk stratification. This limitation is particularly relevant given the well-established prognostic implications of molecular subgroups in adult medulloblastoma. Nevertheless, the clinical course, characterized by early recurrence, rapid neurological deterioration, and extensive neuroaxis dissemination, suggests the presence of high-risk biological features.

Leptomeningeal dissemination through cerebrospinal fluid pathways is a recognized biological feature of medulloblastoma. At diagnosis, dissemination occurs in approximately 10-20% of adults, compared to 20-30% in pediatric populations [3]. At recurrence, dissemination rates increase and may exceed 40% in some reports. Most spinal metastases are leptomeningeal “drop metastases.” In contrast, true intramedullary spinal metastases, defined as tumor infiltration within the spinal cord parenchyma, are exceedingly rare and primarily described in isolated case reports [8,9]. When present, they are typically associated with advanced or recurrent disease and rapid neurological deterioration, reflecting aggressive tumor biology and poor prognosis [10,11].

Suprasellar metastases with optic chiasm involvement are similarly uncommon. Visual impairment in medulloblastoma more often results from increased intracranial pressure or hydrocephalus rather than direct metastatic invasion of the optic pathways [5]. Only a limited number of cases describe chiasmatic or suprasellar metastatic deposits causing direct visual pathway compromise [1,10].

A structured review of previously reported cases indicates that intramedullary spinal metastases and suprasellar dissemination have been described individually in adult medulloblastoma; however, reports documenting their simultaneous occurrence, particularly in the setting of early recurrence, are exceedingly limited.

The coexistence in our patient of posterior fossa recurrence, suprasellar dissemination with optic chiasm encasement leading to bilateral blindness, and intramedullary spinal metastases reflects diffuse neuraxis involvement consistent with advanced Chang stage M3 disease.

Recurrence in adult medulloblastoma remains clinically significant and frequently devastating. Most adult relapses occur outside the posterior fossa, commonly as distant leptomeningeal or spinal dissemination [9]. Early recurrence within one year after completion of definitive therapy is a well-established adverse prognostic factor. In adult series, median progression-free survival ranges from three to five years in standard-risk patients but declines markedly in early relapse. Reported median overall survival after recurrence is often less than 12-18 months, despite multimodal salvage strategies [9].

Although durable disease control can be achieved in a subset of adult patients treated with combined modality therapy, long-term treatment-related morbidity remains substantial. Several studies have reported late complications following craniospinal irradiation and chemotherapy, including neurocognitive decline, endocrine dysfunction, and secondary treatment-related toxicities that may significantly affect long-term quality of life in survivors [10,14]. Additionally, platinum-based chemotherapy regimens may result in significant hematologic toxicity in adults, including high rates of grade 3-4 cytopenias and treatment interruptions, particularly in resource-constrained settings [11]. These considerations underscore the need to balance aggressive oncologic control with long-term functional preservation.

The absence of molecular profiling in this case limits precise risk classification. However, the early relapse within one year, rapid neurological deterioration, and extensive neuraxis dissemination strongly suggest high-risk biological features despite desmoplastic histology [2,12,13]. This case reinforces the contemporary understanding that histopathological classification alone is insufficient to predict clinical behavior in adult medulloblastoma and that molecular profiling has become essential for accurate prognostic stratification and therapeutic decision-making.

Compared with previously reported adult cases, our patient demonstrated an uncommon and severe pattern of dissemination characterized by the coexistence of posterior fossa recurrence, suprasellar metastases with optic pathway involvement, and intramedullary spinal disease, supporting the concept of widespread neuroaxis dissemination. This case has several limitations that should be acknowledged. First, molecular profiling was not available, limiting precise subgroup classification and risk stratification. Second, intramedullary spinal involvement was based on imaging findings without histopathological confirmation. Third, adjuvant chemotherapy was not administered, which may have influenced disease progression. Finally, follow-up imaging was not fully standardized, including the use of CT instead of MRI at six months, which may have delayed early detection of recurrence.

This case emphasizes three key clinical considerations: (1) the necessity of comprehensive neuraxis imaging at diagnosis and during follow-up; (2) heightened vigilance in patients presenting with new neurological deficits despite recent negative imaging; and (3) the critical role of molecular profiling in guiding risk stratification and management decisions. Early recognition of atypical metastatic patterns may facilitate timely intervention, although prognosis in such scenarios remains poor.

## Conclusions

Adult desmoplastic medulloblastoma may exhibit rapid and clinically significant progression despite standard surgical resection and craniospinal radiotherapy. Early recurrence with diffuse leptomeningeal dissemination, including rare intramedullary spinal metastases and suprasellar optic pathway involvement, can occur even in patients initially staged as non-metastatic. This case underscores the limitations of histology alone in predicting clinical course and highlights the importance of molecular characterization, comprehensive neuraxis staging, and strict radiological follow-up. However, the absence of molecular profiling in this case limits definitive risk stratification and should be considered when interpreting these findings. From a clinical perspective, this case supports the need for early and routine MRI-based surveillance of the entire neuroaxis during follow-up in adult patients, even after initial negative staging, as atypical and rapidly progressive dissemination may occur. Heightened clinical vigilance for new neurological symptoms is essential, as relapse may progress rapidly and carries a poor neurological and oncologic prognosis.
